# Monitoring of National Drug Policy (NDP) and its standardized indicators; conformity to decisions of the national drug selecting committee in Iran

**DOI:** 10.1186/1472-698X-5-5

**Published:** 2005-05-10

**Authors:** Shekoufeh Nikfar, Abbas Kebriaeezadeh, Reza Majdzadeh, Mohammad Abdollahi

**Affiliations:** 1 Iran Drug Selecting Committee Secretary, Drug Regulatory Affairs, Deputy of Food & Drug, Ministry of Health & Medical Education, Tehran, Iran; 2 Department of Toxicology & Pharmacology, Faculty of Pharmacy, and Pharmaceutical Sciences Research Center, Tehran University of Medical Sciences, Tehran, Iran; 3 Department of Epidemiology & Biostatistics, School of Public Health and Institute of Public Health Research, Tehran University of Medical Sciences, Tehran, Iran

## Abstract

**Background:**

Pharmaceuticals have made an important contribution to global reductions in morbidity and mortality. To help save lives and improve health, it is important to be sure about equity to access to drugs, drug efficacy, quality and safety, and rational use of drugs, which are standardized National Drug Policy (NDP) objectives. NDP's indicators are useful to evaluate the pharmaceutical system performance in a country. Iran has adapted a National Drug List (NDL). Since management of drug supply in Iran takes place only for drugs that have been selected in NDL and this list is selected by the member of Iran Drug Selecting Committee (IDSC), thus evaluation of IDSC's decision making during last 5 years is an appropriate way to evaluate the implementation of drug supply system in the country.

**Methods:**

To identify strengths and weaknesses of pharmaceutical policy formation and implementation in Iran, four standard questionnaires of the World Health Organization (WHO) were used. To assess the agreement between decisions of IDSC and standardized NDP indicators in the last 5 years (1998–2002), a weighted questionnaire by nominal group technique based on the questions that should be answered during discussion about one drug in IDSC was designed and used.

**Results:**

There is a totally generics based NDP with 95% local production, that provides affordable access to drugs. The system, structures, and mechanisms were in place; however, they did not function properly in some topics. Assessment of 59 dossiers of approved drugs for adding to NDL during last 5 years showed that IDSC's members pay more attention to efficacy, safety, and rationality in use rather than accessibility and affordability.

**Conclusion:**

Revision of drug system in term of implementation of the processes to achieve NDP's objectives is necessary to save public health. Clarification of NDP's objectives and their impact for IDSC's members will result in improvement of the equity in access to pharmaceuticals.

## Background

The enjoyment of the highest attainable standard of health is one of the fundamental rights of every human being without distinction of race, religion, political belief, and economic or social condition [1]. Everyone has the right to a standard of living adequate for the health of himself and of his family, including food, clothing, housing, necessary social services, and medical care [2]. Governments and the international community have an obligation to see the right to health progressively realized which includes the responsibility for prevention, treatment and control of disease; and the creation of conditions to ensure access to health facilities, goods and services [3]. Access to goods and services include of course the provision of essential medicines necessary for the prevention and treatment of prevalent diseases [4]. In addition, access to essential medicines is fundamental to human rights [5].

Pharmaceuticals have made an important contribution to global reductions in morbidity and mortality [6]. In many developing countries, medicines represent the largest household health expenditure. To help save lives and improve health by closing huge gap between the potential that essential drugs have to offer and the reality that for millions of people medicines are unavailable, unaffordable, unsafe or improperly used, World Health Organization (WHO) is already working with a wide range of partners to achieve this aim by drugs and medicines, and by working with countries to ensure equity to access to drugs, drug quality and safety, and rational use of drugs [7]. The National Drug Policy (NDP) process brings all interested parties; legislation/regulation, quality control, local production, education of consumers, prescribers, dispensers, drug evaluation, selection and registration and rational use; together to focus political improvement and also policy guidance, management tools, and training materials, derived from successful drug list initiatives, do exist.

Essential drugs are one of the tools for fighting ill health. By increasing access to essential drugs, their safety and their rational use, we could make the pharmaceutical potential to improve health and save development gains [8].

Essential drugs are high-value commodities. Their availability draws patients to health facilities, where they can also benefit from preventive services. Moreover, if drug procurement is efficient and transparent, the confidence of governments and ministry in the country's health system is increased, and provision of financial and other resources for health system development encouraged [7,9,10].

Since using appropriate drug list has a lot of benefits and impact in health and economic status, it seems that evaluation of this list could be useful for evaluation of country-adapted policies. Iran has already adapted a National Drug List (NDL). This list is selected by Iran Drug Selecting Committee (IDSC). All drug supply management including registration, procurement, inspection, quality control and post marketing control could be done for a drug that it is accepted to be in Iran drug list. Thinking the drug list is one very important element of a NDP, thus analysis and evaluation of the IDSC's decision-making is one necessary part of the process of evaluating the NDP. Therefore, evaluation of IDSC's decision-making during last 5 years may be an appropriate way to evaluate drug supply system in Iran. Actually, these kinds of studies are necessary to assess the organizational and political determinants of the policy process; to help explain the strengths and weaknesses identified by the indicators; and to assist in identifying and assessing strategies to improve pharmaceutical policy implementation.

The main study questions discussed in the study are the followings:

1. Are the existing basic characteristics of the pharmaceutical system as well structures and processes within it ensure achievement of the main NDP goals?

2. Are the criteria used for drug selection by the IDSC for the purposes of NDL creation compatible with the achievement of main NDP goals?

## Methods

In this study, a descriptive, explanatory, and prescriptive objectives methodology has been designed to describe the consequences, stakeholders, interests, and networks involved in the NDP; to help explain how and why a particular decision was reached in the past; and to assist decision-makers in managing the politics of formulation or implementation.

To identify strengths and weaknesses of pharmaceutical policy formation and implementation in Iran, four standard questionnaires that were designed by WHO (Action Program on Essential Drugs) was used (see table 1 for more information). Questionnaires contained four categories of drug policy indicators including background information, structural indicators, process indicators, and outcome indicators. The indicators serve two purposes in the research: assessment of the implementation of NDP by measuring progress in key components (structural and process indicators) and evaluation of the outcomes of NDP (outcome indicators) [11].

**Table 1:** WHO standardized NDP indicators

**Background information**
Population data
Economic data
Health status data
Health system data
Human resources
Drug sector organization
**Structural and process indicators (quantitative and qualitative)**

Legislation and regulation
Essential drugs selection and drug registration
Drug allocation in the health budget/public sector financing policy
Public sector procurement procedures
Public sector distribution and logistics
Pricing policy
Information and continuing education on drug use
**Outcome indicators**

Availability of essential drugs
Accessibility of essential drugs
Quality of drugs
Rational use of drugs

Reference: Brudon–Jakobowicz P, Rainhorn JD, Reich MR: *Indicators for monitoring national drug policies, a practical manual 2*^*nd *^*ed*. Geneva: World Health Organization; 1999.

Background information provides data on the demographic, economic, health and pharmaceutical contexts in which drug policy is being implemented. The information is quantitative data, at a single point in time, which was readily available at the central level. Background information was obtained from Iran statistics center [12], Iran central bank [13], population report of UNICEF [14], health status report of Iran [15], Iran drug statistics annual report [16] and the ministry of health.

Structural indicators provide qualitative information to assess the pharmaceutical system's capacity to achieve its policy objectives. Questions on structural indicators were answered as yes or no based on available information. Process indicators provide quantitative information on the processes by which a NDP is implemented. They assess the degree to which activities are being effectively implemented and the progress over time.

Outcome indicators were used to measure the results achieved and the changes that can be attributed to the implementation of a NDP. They measure the effects of implementation on the overall objectives of NDP including availability and affordability of essential drugs, drug quality, and the rational use of drugs. Outcome indicators were obtained from available information.

In this study, information on structural and process indicators of drug allocation in the health budget and public sector financing policy, public sector procurement procedures, public sector distribution and logistics indicators were collected from the ministry of health, Iran drug statistics annual report [16], drug regulatory affairs, and management and planning organization of Iran. The information of structural and process indicators of drug pricing policy in Iran were collected from ministry of health and drug regulatory affairs information resources. Because of epidemiologic transition and lack of exact prevalence of new pattern of diseases [15] and their standard treatment guidelines in Iran, calculation of the value of the basket of drugs was impossible.

Structural and process indicators of drug allocation in information, continuing education on drug use and rational use of drugs were obtained from Iran drug and poison information center, and rational drug use (RDU)/prescribing auditing committee [17,18].

Selection of drugs for adding to list is performed in IDSC by considering of efficacy and safety, rationality in use, accessibility, and affordability altogether. To assess the agreement between decisions of drug selecting committee and standardized NDP indicators in the last 5 years (1998–2002), an additional weighted questionnaire was designed based on the questions that should be answered about any drug during decision in IDSC. Questionnaire mostly included questions about the history and documentations on efficacy and safety of drugs and also pharmacoeconomics assessment of drugs that are usually provided by applicants. In addition, information related to above fields that are usually obtained from reliable and independent sources by expert team of the secretary of IDSC were included. Additional questions were about identification of applicants and history of clinical trials especially in Iran, approval record of drug by international agencies like FDA, existence of drug standard treatment guidelines (STGs), potential to be produced by local factories, the supportive coverage umbrella like subsidization and insurance, and also price of the nominated drug. This information was designed in a 29-question form with yes/no pattern. The ideal point for questionnaire could be acquiesced with all positive answers except one. Final decision for a new molecule to be added to NDL is performed in IDSC with ten members who are selected and authorized by minister of health. Therefore, questionnaire was weighted by nominal group technique and by 10 members of IDSC who had mostly participated during last 5 years in IDSC meetings. The relation between each question and four indicators of NDP including: "efficacy and safety", "affordability", "availability and accessibility" and "rationality in use" have been asked and in case of each positive relation, the member of IDSC was asked to give a score from 1–5 for that indicator and for negative answer we considered the score of zero for it. Related to the point which obtained from the IDSC's opinion, any "yes" answer for each question could acquire scores in the range of +1 to +5 and the answer "no" got score 0 for each indicator. Fifty-nine drugs during 1998–2002 have been approved by IDSC's members. Their weighted questionnaire was filled out for each medicine separately and the results were reported by means of percentage of agreement.

Distribution of decision makers' point of view is available in related scattergram for interested readers (see additional file 1).

## Results

### Demographic, economic, health and pharmaceutical information

Background information about demographic, economic, health and pharmaceutical contexts in 2002 have been summarized in Table 2. As shown in this table, the population of Iran is more than 66480000 with urbanization rate of 65% and 69 years old life expectancy [12]. GNP per capita in Iran is 1750 $US [14]. Total health budget is 12000 billions Rials. Total health expenditure is 7596 billions Rials and 5684 billions Rials is total drug expenditure (8000 Rials≈1 $US). Data in this table also show that the infant mortality rate is 32.1/100000 and maternal mortality rate is 37/100000. Total number of prescribers and pharmacists are 92548 and 10334 respectively.

**Table 2:** Background information about the demographic, economic, health and pharmaceutical contexts in 2002

**Demographic data**	
Total population	66480000
Average annual growth of the population	1.5%
Rate of urbanization	65%
Life Expectancy	69 years
GNP per capita	1750 **$US**
Average annual rate of inflation	15.8%
Infant mortality rate	32.1/100000
Maternal mortality rate	37/100000
**Pharmaceutical related data**	

Total number of prescribers	92548
Total number of pharmacists	10334
Total health budget	12000 billions Rials (1.5 billions $US)
Total health expenditure	7596 billions Rials (0.9 billions $US)
Total drug expenditure	5684 billion Rials (0.7 billions $US)
Total number of drugs in national drug list	1516
Total number of drugs under generic name sold in the country	1288
Total value of local production (numbers)	96.1% (19.55 billions/20.348 billions)
Total value of drug imports (numbers)	3.9% (797.41 millions/20.348 billions)
Total number of drug manufacturing units in the country	57
Total number of wholesalers in the country	132
Total number of private pharmacies in the country	6100
Total number of private pharmacies in the three major urban areas	1534

8000 Rials≈ 1 $US

Table 2 indicates that 96.1% (19.55 billions/20.348 billions) of drugs in use are produced in local pharmaceutical factories and the total number of drug manufacturing units in the country is 57. The total number of dosage forms in NDL is 1516 for 933 INN names that 1288 of them are marketed by generic name in the country. There are 6100 private pharmacies all over the country. Drug supply management is performed centrally in Drug Regulatory Affairs of MOH including drug selection, registration, procurement, distribution, pricing and subsidization, GMP control, and rational use of medicines.

### Structural and process indicators of legislation and regulation, drug selection and registration

Structural and process indicators of legislation and regulation, essential drug selection, and drug registration in Iran in 2002 have been summarized in Table 3. As shown in this table, NDP has been established in Iran and updated during last 10 years. Regulations have been issued on the basis of drug legislation and there is a drug regulatory authority to control registration, licensing system, the sale and distribution of drugs. Pharmacists are legally entitled to substitute generic drugs for brand name products. There is a quality control section, which carries out required inspections in different stages of pharmaceuticals production. Data show that seventy percents of total number of drug outlets inspected was in contravention but no sanction has been implemented for them. Iran enjoys primary health care system (PHC). About 5000 public health centers are responsible to provide health services to rural and underdeveloped areas including dispensing of about 400 drugs freely. Value of drugs from NDL procured in the public sector, out of total value of drugs procured in the same sector was 27%. Of total number of drugs, 95% was prescribed and sold from NDL. Hundred percent of samples transferred from manufacturers to the laboratory of MOH are tested. Nevertheless, it should be noted that samples are not collected from the market; they are collected directly from manufacturer companies. Table 3 showed that only 910 out of 1516 dosage forms of NDL (60%) are produced locally.

**Table 3:** Structural and process indicators of legislation and regulation, essential drug selection, and drug registration in Iran in 2002

Is there an official national drug policy document updated in the past 10 years?	+
Is there drug legislation updated in the past 10 years?	+
Have regulations based on the drug legislation been issued?	+
Is there a drug regulatory authority whose mandate includes registration and inspection?	+
Is there a licensing system to regulate the sale of drugs (wholesalers, pharmacists,...)?	+
Are pharmacists legally entitled to substitute generic drugs for brand name products?	+
Are there legal provisions for penal sanctions?	+
Is there a check-list for carrying out inspections in different types of pharmaceutical establishments?	+
Are there any institutions where quality control is carried out?	+
Is the WHO Certification Scheme on the Quality of Pharmaceutical Products Moving in International Commerce used systematically?	+
Are there controls on drug promotion based on regulations and consistent with the WHO Ethical Criteria for Medicinal Drug Promotion?	+
Is there a national essential drugs list (EDL)/formulary using INN officially adopted and distributed countrywide?	+
Is there an official drug committee whose duties include updating the national drugs list?	+
Has the national essential drugs list /formulary been updated and distributed countrywide in the past five years?	+
Do drug donations comply with the national drugs list?	+
Are there formal procedures for registering drugs?	+
Is there a drug registration committee?	+
Is drug registration renewal required at least every five years?	+
Number of drug outlets inspected, out of total number of drug outlets in the country.	100% (132/132)
Number of drug outlets in violation, out of total number of drug outlets inspected.	70% (92/132)
Number of sanctions and administrative measures implemented, out of total number of violations identified.	0%
Number of advertisements in violation of regulations on the ethical promotion of drugs, out of total number of advertisements monitored.	10% (5/49)
Number of sanctions implemented for advertisements in violation of regulations, out of total number of violations identified.	100% (5/5)
Value of drugs from the national drugs list procured in the public sector, out of total value of drugs procured in the same sector.	27% (415/1516)
Number of drugs from the national drugs list prescribed, out of total number of drugs prescribed.	95% (1440/1516)
Number of drugs from the national drugs list sold, out of total number of drugs sold.	95% (1440/1516)
Number of locally manufactured drugs sold in the country from the national drugs list, out of the total number of drugs from the national drugs list.	60% (910/1516)
Number of combination drugs newly registered, out of total number of newly registered drugs.	0% (0/243)
Number of registered drugs, which are banned in other countries, out of total number of registered drugs.	0% (0/243)

### Structural and process indicators of financing policy, procurement, distribution, logistics, and pricing policy

Structural and process indicators of drug allocation in the health budget and public sector financing policy, public sector procurement procedures, public sector distribution and logistics, and pricing policy in Iran in 2002 are summarized in Table 4. Existence of international nonproprietary name (INN) system for listing the medicines, and their procurements, distribution, and selling in private pharmacies have been indicated in this table. Data indicate that drug procurement unit receives required foreign currency in less than 60 days from request to release and the average lead-time from drug order to receipt at central level is less than eight months. The total margin used by wholesalers and retailers is less than 35% of the CIF (cost, insurance, freight) price. There is a system for monitoring drug prices regulated in the private sector. The average lead-time required for a sample order in the last year, out of average lead-time during the past three years decreased about 80%. The number of drugs failed from quality control testing out of the number of drugs tested was 13%. The number of drugs beyond the expiry date out of the total number of drugs surveyed was 0.1%.

**Table 4:** Structural and process indicators of drug allocation in the health budget and public sector financing policy, public sector procurement procedures, public sector distribution and logistics, and pricing policy in Iran in 2002

**Budget and pricing**	
Is the drug budget spent per year more than 20% of the MOH operating budget spent per year for the last three years?	+
Is the drug budget spent per capita more than US$1.00 per year for the last three years?	+
Is the drug budget spent for national hospitals less than 40% of the total drug budget spent for the last three years?	-
Has the drug budget spent per capita increased in the last three years?	+
Are there any financing systems in addition to the drug budget that contribute to the provision of drugs in the public sector?	+
Are drug prices regulated in the private sector?	+
Is there at least one major incentive for selling drugs at low cost in the private sector?	+
Is the total margin used by wholesalers and retailers less than 35% of the CIF price?	+
Is there a system for monitoring drug prices?	+
Are essential drugs sold under INN or generic name in private drug outlets?	+
Average time period of payment for a sample of orders, out of average time period of payment stated in contract.	100% (7 days/7 days)
**Procurement**	

Are drugs usually procured in the public sector through competitive tender?	-
Is there a system for monitoring supplier performance?	+
Are procurements done under INN?	+
Does the procurement unit receive foreign currency in less than 60 days (from request to release)?	+
Is procurement in the public sector limited to drugs on the national drugs list?	+
Is the average lead time (from order to receipt at central level) less than eight months?	+
Is procurement based on a reliable quantification of drug needs?	+
Average lead time for a sample of orders in the last year, out of average lead time during the past three years.	80% (7/9)
Number of drugs/batches tested, out of number of drugs/batches procured.	0%
Number of drugs/batches that failed quality control testing, out of number of drugs/batches tested.	13% (147/1132)
Average time between order and delivery from central store to remote facilities in the last year, out of average time between order and delivery in the past three years.	-
**Storage**	

Are good storage practices observed in the central procurement/distribution unit and/or major regional warehouses?	+
Is the information recorded on the stock cards for drugs?	+
Are the stocks for drugs within their expiry dates in the central procurement/ distribution unit and/or major regional warehouses?	+
Have all incoming products been physically inspected the central procurement/distribution unit and/or in the major regional warehouses?	+
Are drugs which are not on the national drugs list in stock in the central procurement/distribution unit and/or in the major regional warehouses?	-
Are 80% or more of the vehicles of the central procurement/distribution unit and/or major regional warehouses in working condition?	+
Are essential drugs sold under INN or generic name in private drug outlets?	+
Number of drugs beyond the expiry date, out of the total number of drugs surveyed	0.1% (2/1400)

### Structural and process indicators of RDU

Structural and process indicators of drug allocation in information and continuing education on RDU in Iran in 2002 is presented in Table 5. This table shows that the national drug information book has been regularly published and revised within the past five years. There is an official continuing education system on RDU for prescribers and dispensers. There are drug information centres that provide regular information on drugs to prescribers and dispensers. Data of table 5 also show that there is a national therapeutic guide with standardized treatments but the concept of essential drugs is not part of the curricula in the basic training of health personnel and there are not therapeutic committees in the major hospitals. There is at least one injection in 47% of total prescriptions surveyed with average number of 3.6 drugs prescribed for each patient. Antidiarrheal drugs have been prescribed for children fewer than five in 19% of cases for treatment of diarrhea. Average retail price of standard treatment of pneumonia out of the average retail price of a basket of food is 21%. No budget has been devoted for enlightening public on RDU. Of prescribers surveyed, only 7.6% had direct access to a national drug formulary. Only 6.9% of prescribers received independent drug bulletins sent by drug and poison information centres. Number of training sessions on RDU for prescribers in the last year out of the average number of training sessions organized in the past three years was 13%. Number of prescribers who attended at least one training session in the last year, out of total number of prescribers surveyed was 50%.

**Table 5:** Structural and process indicators of drug information, continuing education on drug use, and rational use of drugs in Iran in 2002

Is there a national publication (formulary/bulletin/manual, etc.), revised within the past five years, providing objective information on drug use?	+
Is there a national therapeutic guide with standardized treatments?	+
Is the concept of essential drugs part of the curricula in the basic training of health personnel?	-
Is there an official continuing education system on rational use of drugs for prescribers and dispensers?	+
Is there a drug information unit/centre?	+
Does the drug information unit/centre (or another independent body) provide regular information on drugs to prescribers and dispensers?	+
Are there therapeutic committees in the major hospitals?	-
Are there public education campaigns on drug use?	+
Is drug education included in the primary/secondary school curricula?	+
Number of prescribers having direct access to a (national) drug formulary, out of total number of prescribers surveyed.	7.6% (7034/92548)
Number of training sessions on drug use for prescribers in the last year, out of average number of training sessions organized in the past three years.	13% (120/924)
Number of prescribers who have attended at least one training session in the last year, out of total number of prescribers surveyed.	50% (15203/32524)
Number of issues of independent drug bulletins published in the last year, out of average number of issues of independent drug bulletins published per year in the past three years.	0% (0/13)
Average number of copies of independent drug bulletins sent to prescribers, out of total number of prescribers.	6.9% (6386/92548)
Amount spent on public education campaigns on drug use, out of total amount spent on public health education campaigns.	0% (0/300000 $US)
Average retail price of standard treatment of pneumonia, out of the average retail price of a basket of food.	21% (2.9/13.75 $US)
Average number of drugs per prescription.	3.6
Number of prescriptions with at least one injection, out of the total number of prescriptions surveyed.	47.4% (692530/1461033)
Number of children under five with diarrhea receiving antidiarrheal drugs, out of the total number of children under five with diarrhea surveyed.	19% (266/1400)

### IDSC's function

**Figure 1:**
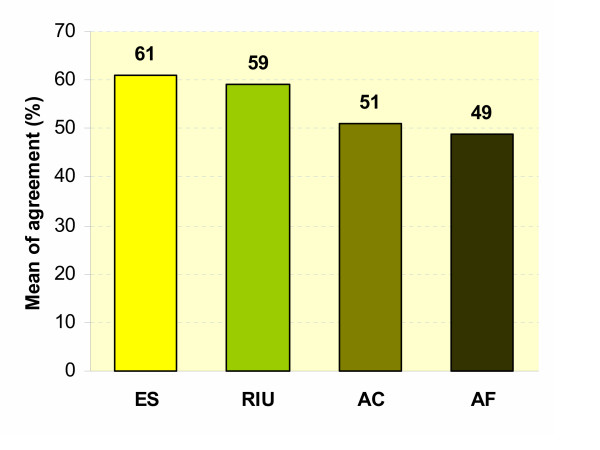
**Estimation of NDP indicators including efficacy and safety, affordability, availability and accessibility, and rational use of drugs on approved drugs by IDSC during 1998–2002. **Number of evaluated drug dossier is 59. ES means efficacy and safety. RIU means rationality in use. AC means accessibility and AF means affordability.

Estimation of NDP indicators including efficacy and safety, affordability, availability and accessibility, and RDU on approved drugs by IDSC during 1998–2002 is shown in Figure 1. The assessment of 59 dossiers of approved drugs for adding to NDL showed that IDSC's members pay more attention to efficacy, safety, and rationality in use of drugs rather than accessibility and affordability.

## Discussion

Pharmaceuticals have made an important contribution to global reductions in morbidity and mortality [6]. Drug supply management has a great impact in health services, thus, evaluation, redeveloping and implementation of drug policy has an important role in health system. Policy guidance, management tools, and training materials are derived from a successful drug list initiative [7]. IDSC is responsible to establish NDL. In this study, Iran drug policy indicators were monitored by four standardized questionnaires of WHO [11].

### Demographic, economic, health and pharmaceutical information

As shown in table 2, the population of Iran is more than 66480000 with urbanization rate of 65% and 69 years old life expectancy [12]. GNP per capita in Iran is 1750 $US which 5.7% of that, is usually spent for health expenditures [13]. Related to demographic, economic, health, and pharmaceutical contexts, Iran is one of the developing countries [14]. The percentage of products in NDL which are produced locally measures a country's self sufficiency for supplying the most essential pharmaceutical products [10]. Table 2 indicates that 96.1% of drugs are produced in 57 local pharmaceutical factories. Production of drugs domestically decreases the final price of drugs and thus makes them more affordable and presents more feasible and reliable procurements.

### Legislation and regulation

As shown in table 3, NDP has been established in Iran and updated in the last 10 years with inclusion of necessary regulations and drug control legislations. By regulation of national medical council, only drugs exist in NDL should be prescribed [19]. However, this regulation has not been implemented absolutely. About 70% of total number of drug outlets inspected had problem of quality that may negatively affect objectives of NDP [7]. There was no special rule to sanction them at that duration of time but it has now being established. Drug control and regulations are measures of a government's capacity to implement beneficial policies and practices in pharmaceutical management. If a government does not enforce legislation and regulation, it means a situation where plans for pharmaceutical improvement exist only on paper and not in reality [10]. Moreover, even if drugs are available, weak drug regulation could mean that they are substandard, thus spending on these medicines seems to be the major source for poverty [8]. Almost all data have been feedback to policy makers and fortunately, improvements are observable in some parts. In addition, some items have been taken in the priority of many relevant committees to find reasonable resolutions.

### Access issues

Access to drugs is a key priority. From the consumer's point of view, access means that drugs can be obtained with reasonable traveling distance from health facilities [7].

### Physical access

Appropriate distribution network could provide good accessibility for medicines within the country. Existence of INN system for listing the medicines, and their procurements, distribution, and selling in private pharmacies are the strengths of Iran NDP (table 4) [6,10,11,20]. Private pharmacies belong to pharmacists who are allowed to establish their pharmacies under district regulation and legislation of MOH. They are responsible to provide medicines of NDL for all patients with fixed price which announced by MOH. They have to procure their medicines from distribution centers. These centers work under supervision of government. They procure medicines from 57 local pharmaceutical factories and 3 important drug procurement centers. Procurement of drugs from local industries is in priority and other pharmaceuticals import from other countries. It should be noted that INN system listing existing in Iran is not against the availability of patent drugs. If the patent drug is approved in NDL, it would be subsidized too. A specific guideline has been established in Iran for availability of drugs that are not included in NDL. According to this guideline, the physician who prescribe a non-NDL drug should sign an agreement and accept the responsibility for any possible unwanted effects that might happen by use of that drug in that specific patient. The other point is that the physician should convince the drug regulatory affairs about not effectiveness of the existed similar drugs in the NDL. Finally, the drug will be imported for that patient without any subsidy or insurance coverage only in amount of prescription [19].

### Pricing

Information in table 4 also indicate the existence of a good structure and process for financing in drug supply leading to better affordability. Potential for local production of drugs could result in constant accessibility of drugs with suitable prices [9,10]. It is considerable to note that in Iran, there is a powerful subsidizing system for drugs. Insurance companies pay about 80% of drug price, which diminishes patient's out of pocket spending.

### Evaluation of rational drug use

The evaluation of RDU in Iran is presented in table 5. RDU means the promotion of therapeutically sound and cost-effective use of medicines by health professionals and consumers [7,8]. Iran was very successful in establishing of more than 20 drug and poison information centers around country since first 1997 and providing national independent drug information bulletins [17] to promote RDU. It is interesting to note that most of "standard treatment guidelines" about communicable diseases in Iran have been written [21]. Concerning epidemiologic transition [15], it is important to provide guidelines for non-communicable illnesses specially diabetes and cardiovascular diseases as well as infectious diseases. Lack of drug and treatment committee's in hospitals is indicated in Table 5. To ensure proper use of drugs in therapeutically sounds and cost-effectiveness way, integrated approaches between medicines and treatment management are required [22]. There are evidences for irrational use of drugs in Iran such as existence of at least one injection in 47% of total prescriptions surveyed and average number of 3.6 drugs prescribed for each patient or receiving antidiarrheal drugs for children under five years old (20%) in treatment of diarrhea [10,21,23]. On the other hand, no budget has been devoted for cultivating public on RDU [6,10,11] that can be a deficit for drug policy makers.

### Function of IDSC

Essential medicines are perhaps the most cost-effective element of public health after immunization [20,24]. Regarding figure 1, the most interested factor for IDCS members were efficacy and safety of drugs (61%). This item is mostly experience-based and could be found in many classic pharmacological and clinical references. However, the existence of safe and effective medicines could save millions of lives and prevent untold suffering all over the world but is not enough in term of RDU [7]. In decision making of IDCS, information obtained about experience of drug usage or approval in abroad has been very helpful. In addition, IDSC pay enough attention to viewpoints of health professional societies. Results of clinical trials have also good impact in decision-making by IDSC members. Worldly, the criteria for new drug approval have been developed from experience-based to evidence-based approach. In this regard, WHO has done many efforts to encourage countries to provide evidence-based NDL to implement RDU. Evidence used by WHO to add or remove a drug might provide some basis in country-level decision-making, but in some cases, local trials should be carried out. It should not be forgotten that existence of reliable and independent information on questioned drug [25] especially on its costs [26] are important elements for decision-making in IDSC. Accordingly, holding adequate training workshops for IDSC members to teach evidence-based medicine seems necessary [22].

RDU was the second frequently considered factor by decision-makers of IDSC. Existence of approved treatment guidelines for a drug before submitting the application to IDSC is very important. Based on the criteria examined in the present study, only 59% of drugs were selected according to therapeutic guidelines in the last 5 years. To promote RDU, it is necessary to provide updated STGs for new patterns of different diseases like cardiovascular or cancer. WHO emphasizes to provide essential life-saving drugs developed for leading non-communicable diseases as well as leading infectious ones [5,7,20]. Every one of these diseases impinges detracting from health gains and delaying progress in other areas such as education and economic development [7,27]. Since 1999, WHO has recommended countries to design their NDL according to national STGs [25].

Accessibility and affordability with means of 51% and 49% respectively also support that IDSC's members have not paid enough attention in this respect. The price of pharmaceuticals seems a substantial barrier to access for governments and health insurers [24]. An important point is that cost-effectiveness has been almost a forgotten element in decision-making during the last 5 years (1998–2002). Considering financial patterns in providing pharmaceuticals is very important element, and if not, it will face countries with the problem of drugs procurement [28]. Recently two special workshops on pharmacoeconomics were held for IDSC members and stakeholders. This can be a starting point to show that importance. After this study and finding these interesting data, new questionnaires for drug selecting has been launched and implemented to improve the process of drug selection by considering standardized indicators of NDP.

Finally it should be reminded that essential medicines are those that satisfy the priority health care needs of the population. They are selected with due regard to public health relevance, evidence on efficacy and safety, and comparative cost-effectiveness. Essential medicines are intended to be available within the context of functioning health systems at all times in adequate amounts, in the appropriate dosage forms, with assured quality and adequate information, and at a price that the individual and the community can afford. The implementation of the concept of essential medicines is intended to be flexible and adaptable to many different situations; exactly which medicines are regarded as essential remains a national responsibility [29].

Revision of drug system in Iran for implementation and improvement of the processes to achieve NDP's objectives is necessary to save public health. Clarification of NDP's objectives and their impact for IDSC's members will be helpful to improve the equity in access to pharmaceuticals.

## Conclusion

Finally, it is possible for us to answer to two main questions of this study as mentioned in introduction. Overall results of tables 2,3,4,5 show that in Iran like most developing countries, the system, structures, and mechanisms are in place, however, they often do not function properly, which impeded implementation of strategies and policies and achievement of objectives [6]. In addition, data of figure 1 shows that the criteria used for drug selection by IDSC are 50–60% compatible with achievement of main NDP goals. Collectively, it is concluded that drug system in Iran is in place but needs some revisions. Further studies are required to evaluate the exact impact of drug supply management in health in Iran.

## Competing interests

This study was the outcome of the MPH thesis of SN and was supported by a grant from Tehran University of Medical Sciences.

## Authors' contributions

SN carried out all parts of the study (designing, conducting and writing the manuscript). AK and RM participated in design and coordination of the study. MA participated in designing, and writing the manuscript. All authors read and approved the final manuscript.

## Pre-publication history

The pre-publication history for this paper can be accessed here:



## Supplementary Material

Additional File 1 The scattergram of decision makers' point of view. The relation between each question and four indicators of NDP including: "Efficacy and Safety", "Affordability", "Availability and Accessibility" and "Rationality in use" have been asked and in case of each positive relation, the member of IDSC was asked to give a score from 1–5 for that indicator and for negative answer we considered the score of zero for it. Related to the point which obtained from the IDSC's opinion, any "yes" answer for each question could acquire scores in the range of +1 to +5 and the answer "no" got score 0 for each indicator. Their weighted questionnaire was filled out for each member separately and the results were reported by means of percentage of agreement. Click here for file
