# Supplementary figures and images for: Monitoring of National Drug Policy (NDP) and its standardized indicators; conformity to decisions of the national drug selecting committee in Iran
Source: BMC Int Health Hum Rights. 2005 May 10;5:5. doi: 10.1186/1472-698X-5-5 (PMC1145184; doi:10.1186/1472-698X-5-5)

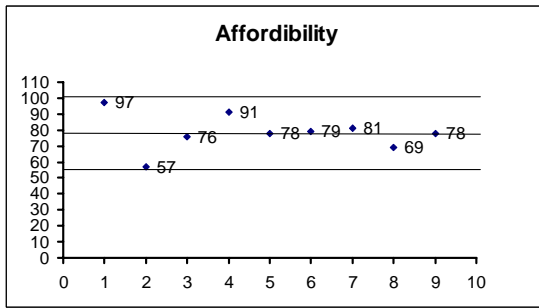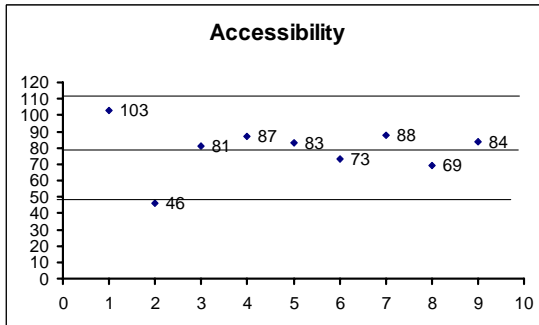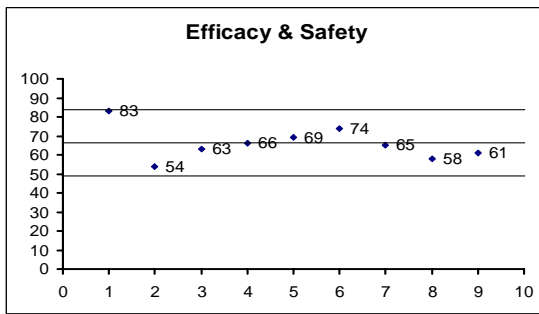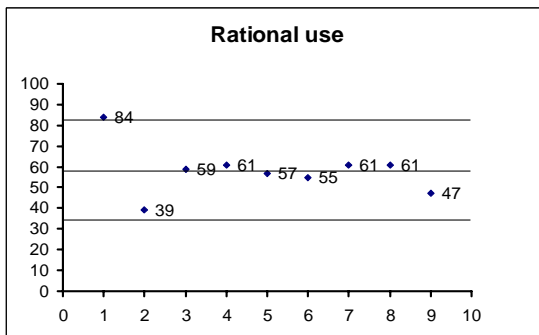

Supplement: Additional File 1 — The scattergram of decision makers' point of view. The relation between each question and four indicators of NDP including: "Efficacy and Safety", "Affordability", "Availability and Accessibility" and "Rationality in use" have been asked and in case of each positive relation, the member of IDSC was asked to give a score from 1–5 for that indicator and for negative answer we considered the score of zero for it. Related to the point which obtained from the IDSC's opinion, any "yes" answer for each question could acquire scores in the range of +1 to +5 and the answer "no" got score 0 for each indicator. Their weighted questionnaire was filled out for each member separately and the results were reported by means of percentage of agreement. [file 1472-698X-5-5-S1.pdf]
